# The 2026 western US snow drought was about four times more likely due to climate change

**DOI:** 10.1073/pnas.2612961123

**Published:** 2026-07-20

**Authors:** Adrienne M. Marshall, Marianne Cowherd, Stefan Rahimi, Yuhong Ye

**Affiliations:** ^a^ Hydrologic Science and Egineering Program, Colorado School of Mines, Golden, CO 80401; ^b^ Department of Earth Sciences, Montana State University, Bozeman, MT 59717; ^c^ Department of Atmospheric Science, University of Wyoming, Laramie, WY 82071

**Keywords:** snow drought, climate change, extreme event attribution, western US, water resources

## Abstract

In the spring of 2026, anomalously low snow conditions in the western United States threatened winter recreation and water supplies. Here, we investigate: to what extent was this snow drought attributable to the climate change that has occurred since the pre-industrial period? We find that a snow drought this severe across the western United States was approximately 4.4 [95% CI: 2.6, 9.4] times more likely in the current climate than in the preindustrial period. In the Upper Colorado River Basin, the snow drought was approximately 14 times more likely [0.09, 4,300]. Given a projected increase in the frequency of snow droughts at least this severe in a moderately high emissions warming scenario, the lived experience of this event may help scientists, resource managers, and the public consider what western US snow might look like if greenhouse gas emissions are not aggressively reduced.

In the winter and spring of 2026, a snow drought gripped the American West; “record-low” conditions were widely reported (1). The snow drought was associated with anomalously high temperatures, with moderately dry to average precipitation (2). While it is still too early to know how the consequences of this snow drought will materialize, the US Bureau of Reclamation projected for the first time in spring 2026 that Lake Powell would fall below its minimum power pool threshold by the following winter in median climatological conditions (3). The snow drought is also likely to increase fire risk (4) and reduce water availability for energy (5), agriculture (6), and municipalities.

As the snow drought progressed, scientists, the recreation industry, and the press asked whether the low snow anomaly was attributable to climate change (7). The frequency, severity, and temperature of snow droughts increase in climate models (8, 9) but formal climate attribution has only rarely been applied to snow drought [see Mote et al. (10) for one exception]. Since then, the science and data available for climate attribution studies have advanced substantially (11). Here, we ask: to what extent is the 2026 western US snow drought attributable to climatic changes that have occurred since the preindustrial period?

## Methods

We take a probabilistic approach to assessing the role of climate change in the 2026 snow drought across the western United States. We obtained volumetric snow water equivalent (SWE) from the 0.1° (9-km) ERA5-Land product (12) on March 15, 2026, and on March 15 throughout the ERA5-Land period of record (1950-present). March 15 was selected because it represented approximate peak SWE. After ensuring that ERA5-Land adequately represents the 2026 SWE anomaly, we assess the probability of occurrence of the 2026 snow drought in the current vs preindustrial climate periods. To do so, we use 10 ensemble members from the CESM2-LE project (13) that were previously dynamically downscaled to a 9-km resolution over the historical and SSP3-7.0 scenarios (14). We fit generalized extreme value distributions of SWE volume z-scores over a preindustrial period (1850-1900) and the 21-y period encompassing 2026 (2016-2036). We estimate the likelihood of the 2026 snow drought in the current (“factual”) period and the preindustrial (“counterfactual”) period, dividing the factual by the counterfactual probability to obtain a risk ratio (RR). We further assess how the risks of such an event would evolve over the 21st century in a moderately high emissions warming scenario (*SI Appendix*, *Supplemental Methods*; data in (15)).

## Results

**Fig. 1.**
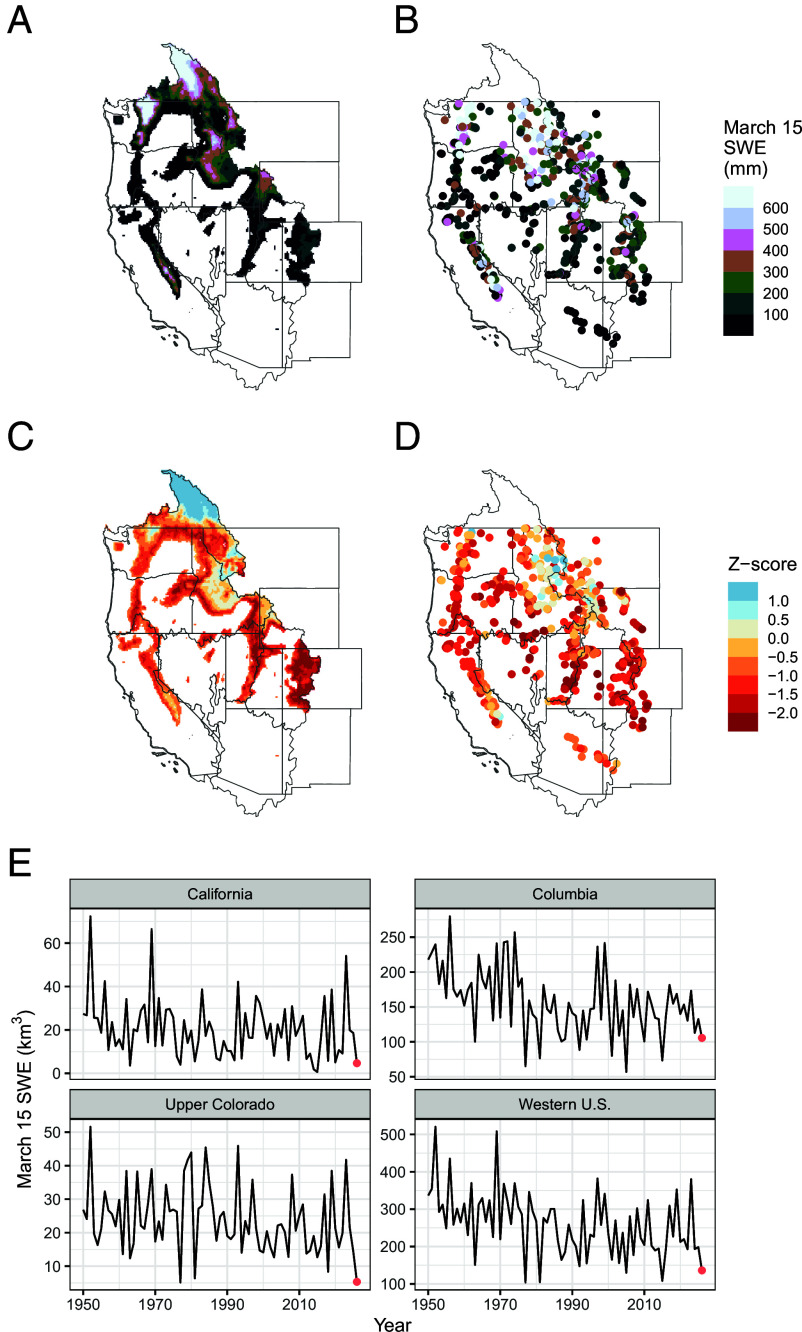
Maps of (*A* and *B*) March 15 SWE and (*C* and *D*) the z-score relative to 1987-2026. The *Left* column shows ERA5-Land SWE; the *Right* column shows in situ SWE from the Snow Telemetry (SNOTEL) network and California Department of Water Resources snow pillows. (*E*) ERA5-Land March 15 SWE over major HUC-2 regions of the western United States.

The 2026 snow drought was widespread and severe (Fig. 1). At 17% of snow pillows and 20% of ERA5-Land pixels, the observed March 15 SWE was the lowest over the 1987-2026 period. It was at or below the 10th percentile for 45% of snow pillows and 50% of pixels. Over the 77-y period of record in ERA5-Land volumetric SWE was low, although not record-breaking, in all spatial domains (Fig. 1*E*).

**Fig. 2.**
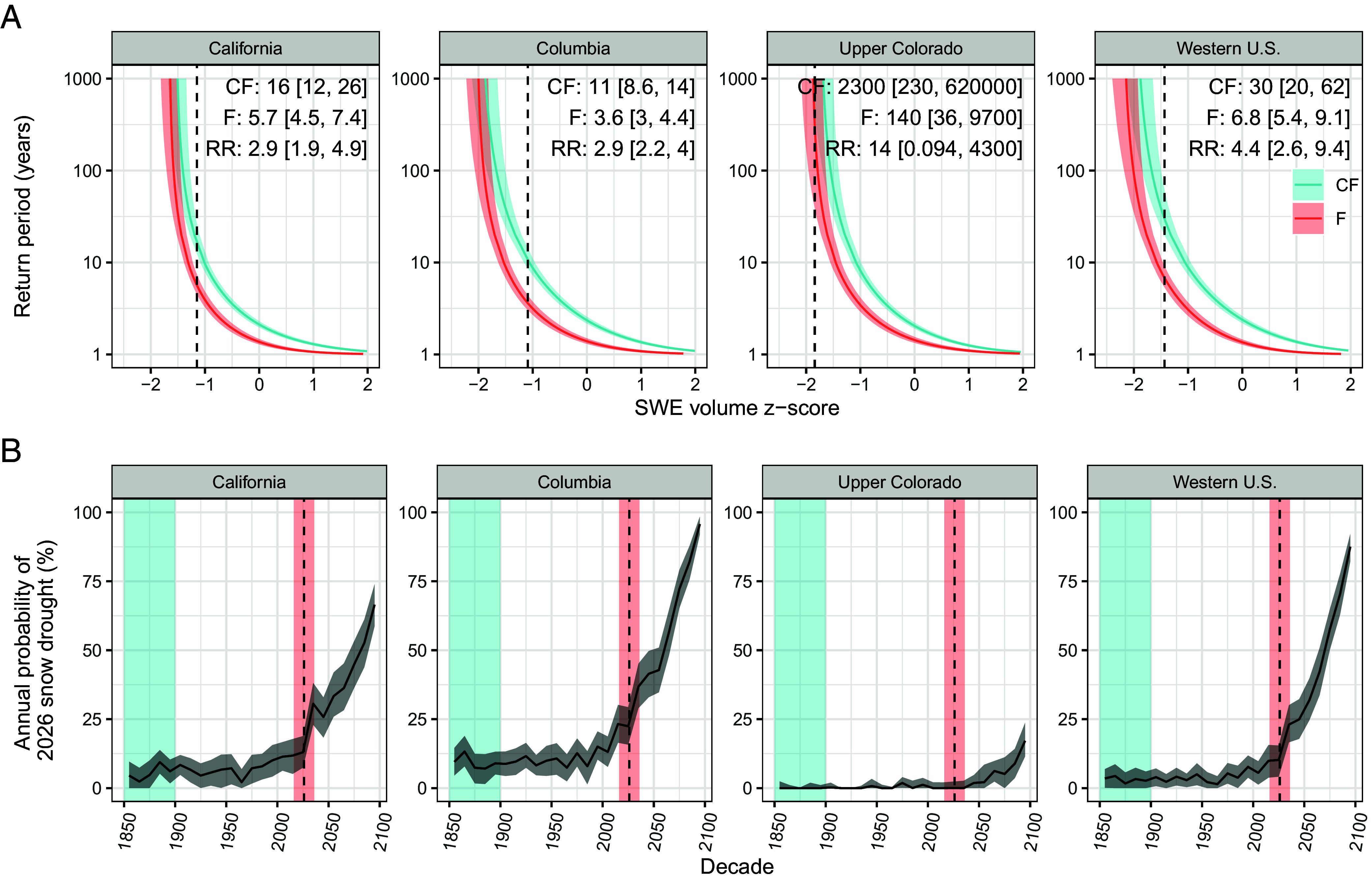
(*A*) SWE volume z-scores and associated return intervals for each spatial domain in the counterfactual (CF) and factual (F) periods. The red vertical line shows March 15, 2026 SWE volume z-score. Text shows CF and F return intervals, RR, and associated 95% CI. (*B*) Annual probability of a snow drought at least as extreme as that observed in 2026. The vertical dashed line marks 2026; red and blue bars mark the F and CF periods.

Across the western United States, the 2026 snow drought had 6.8 [95%CI:5.4,9.1] year return interval with respect to the current climatology based on the downscaled climate data, and a 30 [20, 62] year return interval with respect to the 1850-1900 counterfactual period (Fig. 2*A*). This results in a median RR of 4.4 [2.6, 9.4], meaning that the 2026 snow drought was approximately four times more likely in current climatology than in 1850-1900. Western US SWE volume was about 40 km^3^ lower (25% smaller) than an event with a comparable return interval in 1850-1900. In the Columbia River Basin and California, median RRs were both equal to 2.9. In the Upper Colorado River Basin, the median RR was 14 [0.09, 3,400], with a wide CI due to very low event probabilities in the counterfactual. Upper Colorado SWE volume was about 2.6 km^3^ lower (33% smaller) than an event with a comparable return interval in 1850-1900.

In the SSP3-7.0 scenario, the probability of a snow drought as severe as that observed in 2026 would increase substantially over the 21st century (Fig. 2*B*). For context, the annual probability of occurrence of western US SWE at or below the level observed in 2026 is 10% [4,15] in the 2020s. This probability more than triples by the 2050s, reaching 32% [24,40], and grows to 87% [82,92] by the 2090s, meaning that a snow drought like the one observed in 2026 would be much more common than not by the late century.

The trajectories of these probabilities differ among major river basins; the patterns of increase in the Columbia River Basin and California are qualitatively similar to that described for the western United States. The Upper Colorado River Basin retains a much smaller probability of occurrence than the western United States even into the 2090s. However, this is not due to a lack of snow decline. Instead, the 2026 snow drought was so severe in the Upper Colorado that its probability in the 2020s was only 0.02% [0,2.3]. The probability rises to 2.1% [0,8.4] by the 2050s and to 17% [11,24] by the 2090s, but such a severe snow drought does not become the norm, even by the end of the century.

## Discussion

Across western US basins, the snow drought conditions observed in March of 2026 were severe, buoyed only partially by positive SWE anomalies in the upper reaches of the Columbia River Basin. In today’s climate, snow drought conditions this severe are approximately four times more likely than they would have been in the preindustrial climate. The 40 km^3^ magnitude of missing snow relative to a similar return-interval event in 1850-1900 is comparable to the 35 km^3^ storage capacity of Lake Mead, the region’s largest reservoir. In the Upper Colorado River Basin, conditions were about 14 times more likely in our current climate, with the uncertainty range reaching into conditions so extreme that probabilities are difficult to estimate with confidence. While there is uncertainty in the exact magnitudes of the probabilities and risk ratios, all evidence in this analysis suggests that the 2026 snow drought was vastly more likely in the modern climate than the preindustrial, with additional uncertainty in the Upper Colorado River.

Future work should evaluate other snow droughts, use multiple information streams, use “natural” scenarios rather than the preindustrial period, and assess how risk ratios evolve seasonally. In 2026, the observed record-breaking March heatwave would likely have yielded even more extreme risk ratios by April 1. It would also be useful to attribute the warm and dry anomalies associated with snow drought events, as well as the synoptic-scale drivers: The 2026 event was driven by both thermodynamics and a strong seasonal dipole, with anomalous ridging (troughing) across the western (eastern) United States. Finally, attribution of not only extreme events but also their impacts on people and ecosystems is increasingly possible (16) and could be used to identify the extent to which the consequences of snow drought are ultimately attributable to anthropogenic climate change.

One promise of attribution science is that it provides scientists, policymakers, resource managers, and the public with an understanding of climate risks grounded in observed extreme events. Our analysis suggests that in an SSP3-7.0 scenario, 2026-like snow droughts will become more common than not by the late 21st century. This may help resource managers envision a plausible future and the adaptations it requires. We emphasize that these projections are not predictions of the future, but a warning of our best estimates of expected impacts in a moderately high emissions world. Lower emissions scenarios would reduce this risk and should be pursued ambitiously.

## Supplementary Material

Appendix 01 (PDF)

## Data Availability

Data and code data have been deposited in Zenodo (DOI: 10.5281/zenodo.19558205) (15).
