# Supplementary material for: The 2026 western US snow drought was about four times more likely due to climate change
Source: Proc Natl Acad Sci U S A. 2026 Jul 20;123(30):e2612961123. doi: 10.1073/pnas.2612961123 (PMC13416093; doi:10.1073/pnas.2612961123)
Supplement: Supplementary file 1 — Appendix 01 (PDF) [file pnas.2612961123.sapp.pdf]

PNAS

Supplemental Methods for:

The 2026 western U.S. snow drought was five times more likely due to climate change

Adrienne M. Marshall[1], Marianne Cowherd[2], Stefan Rahimi[3], Yuhong Ye[3]

1. Colorado School of Mines, Golden CO 80401
2. Montana State University, Bozeman MT 59717
3. University of Wyoming, Laramie WY 82071

* Adrienne M. Marshall
Email: adriennemarshall@mines.edu

Author Contributions: AMM, MC, and SR developed the methodology for the study. AMM and YY conducted data analysis. AMM wrote the initial manuscript draft, and all authors contributed to text revisions.

Competing Interest Statement: The authors have no competing interests to report.

Classification: Physical Sciences; Earth, Atmospheric, and Planetary Sciences

Keywords: snow drought, climate change, attribution, western U.S.

This file includes:

Main Text
Figures 1 to 2

This file includes:

Supplemental Methods

Supplemental Methods

1 Observational data

We obtained SWE data from in situ snow observations and a gridded reanalysis product (ERA5-Land) to assess the observational record and suitability of ERA5-Land for analyzing the 2026 snow drought. In-situ snow observations were obtained from the Natural Resources Conservation Service (NRCS), including data from the Snow Telemetry (SNOTEL) network and the California Department of Water Resources. We identified sites that had 40 years of March 15 SWE data over 1987-2026, resulting in 659 unique stations.

ERA5-Land offers several strengths for this analysis due to its period of record and resolution: it dates back to 1950 and is globally available with a 9-km resolution. The 9-km resolution, while coarse relative to many other western U.S. products, is comparable to best-available regional downscaled climate products. Further, it has a sufficiently long period of record for the present study. While this coarse resolution limits its skill with respect to the fine-scale spatial distribution of SWE captured by airborne lidar, ERA5-Land is skillful at estimating basin-average SWE when evaluated against airborne lidar (1, 2). We calculated volumetric SWE from ERA5-Land on the first and fifteenth of each month from January 1 through April 1 for the Upper Colorado and Columbia River Basins and major California watersheds draining the western slope of the Sierra Nevada and Cascades, using the HUC-2 boundaries delineated in the USGS Watershed Boundary Dataset. We also summed ERA5-Land SWE over all western U.S. basins on the western side of the Continental Divide.

March 15 was selected as the target of analysis based on an evaluation of ERA5-Land SWE over the course of the 2026 water year across the western U.S. Among data analyzed for the first and 15th of each month, March 15 was the highest, making its analysis approximately equivalent to the commonly used peak SWE. Furthermore, rapid melt later in March 2026 led to low April 1 SWE values and would have conflated the low-peak-SWE signal with the early-melt signal.

We compared magnitudes of March 15 SWE from in situ data and ERA5-Land, calculating SWE and a z-score by subtracting the mean and dividing by the standard deviation. The spatial patterns and magnitudes of the SWE anomaly in these two datasets were well-aligned, and reflected an unusually severe and widespread snow drought. The alignment between ERA5-Land and in situ observations provided confidence that ERA5-Land adequately represented the snow drought event in question, and we could therefore take advantage of its desirable features for an attribution study: a long period of record, capacity to provide volumetric estimates across a large area, and scale alignment with the climate model data ensemble available for factual and counterfactual analysis.

2 Modeled SWE and model adequacy

We used a downscaled data product that is part of the widely used Western United States Dynamically Downscaled Dataset (WUS-D3) product (3, 4). WUS-D3 provides bias-corrected, dynamically downscaled SWE estimates from nine GCMs over 1980-2100 in the SSP3-7.0 scenario. The product used here is 10 ensemble members from the CESM2 large ensemble, previously applied in (5). These were bias-corrected and downscaled in a similar manner to the primary WUS-D3 product (4), but run from 1850-2100, providing access to spatially resolved models of 10 ensemble members over the pre-industrial period (1850-1900).

We assessed model adequacy with respect to ERA5-Land SWE. For each spatial domain (HUC-2s and the western US), we fit a generalized extreme value (GEV) distribution over the ERA5-Land period of record for March 15 SWE, using L-moments to fit the location, scale, and

shape parameters with the extRemes R package (6). We extracted the 95% confidence intervals for these parameters. We then fit a GEV to the WUS-D3 data over the same period of record to determine whether the WUS-D3 GEV parameters fell within the 95% CI of the ERA5-Land GEV parameters. This is an established method of model adequacy evaluation (7). We found that this test was not met in all spatial domains, so we calculated the z-score of volumetric SWE on March 15 in both ERA5-Land and WUS-D3 as follows:

$$z = \frac{x - \mu_x}{\sigma_x} \tag{1}$$

where z is the z-score; *x* is the volumetric SWE value, μ_x is the mean and σ_x is the standard deviation SWE value over the ERA5-Land SWE period of record. We repeated the model adequacy test and found that the WUS-D3 GEV parameters were within the 95% CI of the ERA5-Land GEV parameters for all spatial domains when z-score transformation was applied.

3 Attribution analysis

We define a counterfactual period as 1850-1950, and the factual period as within ±10 years of the 2026 event. We evaluated volumetric SWE trends over the counterfactual period using the Mann-Kendall test to ensure there was no trend in the simulated SWE, and found no statistically significant trends (setting $\alpha = 0.05$) in any HUC2 or across the western U.S. domain. We fit a GEV distribution to the z-score-transformed volumetric SWE data for each counterfactual and factual period. In each case, we captured the extremes of low values by fitting the distribution to the negative of the z-score-transformed data, then calculated the probability of observing an event at least as extreme as the observed snow drought as follows:

$$F(z) = 1 - e^{-1+\left(\frac{\xi(-z-\mu)}{\sigma}\right)^{(-1/\xi)}} \tag{2}$$

where F(z) is the probability of observing a snow drought at least as severe as the observed event *z*, μ is the fitted location parameter, σ is the fitted scale parameter, and ξ is the fitted shape parameter. We fit these distributions for each counterfactual and factual period separately. Risk ratios were calculated as the probability of occurrence in the factual period divided by the probability of occurrence in the counterfactual period. 95% confidence intervals of the probabilities and risk ratios were assessed by bootstrapping (resampling with replacement) N = 5000 times for each reported attribution value. The factual and counterfactual probabilities and risk ratios were obtained for each sample, and confidence intervals were obtained based on the 2.5th and 97.5th percentile values for each distribution. Return intervals were calculated as one divided by the event probability. Return intervals and risk ratios are reported to only two significant digits to reflect uncertainties throughout the analysis pipeline.

4 Projections in future context

To assess how the probability of observing an event at least as severe as the 2026 snow drought would evolve over the 21st century in an SSP3-7.0 scenario, we re-fit the GEV for each decade of the WUS-D3 data (1850-2100) for each spatial domain (100 samples in each GEV). Then we estimated the probability of the 2026 snow drought given the fitted GEVs in each decade, using the same bootstrapping approach as above to assess 95% confidence intervals.

Supplemental References

1. L. Mudryk, C. Mortimer, C. Derksen, A. Elias Chereque, P. Kushner, Benchmarking of snow water equivalent (SWE) products based on outcomes of the SnowPEx+ Intercomparison Project. *The Cryosphere* **19**, 201–218 (2025).

2. E. Ritchie, *et al.*, Benchmarking Catchment-Scale Snow Water Equivalent Datasets and Models in the Western United States. *EGUsphere* 1–38 (2025). https://doi.org/10.5194/egusphere-2025-5514.

3. S. Rahimi, *et al.*, Evaluation of a Reanalysis-Driven Configuration of WRF4 Over the Western United States From 1980 to 2020. *J. Geophys. Res. Atmospheres* **127**, e2021JD035699 (2022).

4. S. Rahimi, *et al.*, Understanding the Cascade: Removing GCM Biases Improves Dynamically Downscaled Climate Projections. *Geophys. Res. Lett.* **51**, e2023GL106264 (2024).

5. A. Dixit, *et al.*, High-resolution models better simulate historical snowpack declines in the Upper Colorado River Basin. *Environ. Res. Lett.* **21**, 054012 (2026).

6. E. Gilleland, R. W. Katz, **extRemes** 2.0: An Extreme Value Analysis Package in *R*. *J. Stat. Softw.* **72**, 1–39 (2016).

7. Committee on Extreme Weather Events and Climate Change Attribution, Board on Atmospheric Sciences and Climate, Division on Earth and Life Studies, National Academies of Sciences, Engineering, and Medicine, *Attribution of Extreme Weather Events in the Context of Climate Change* (National Academies Press, 2016).
